# Chloroform

**Published:** 1871-02

**Authors:** 


### CHLOROFORM.

The increasing mortality from Chloroform is evident from the numerous cases reported in the various Journals. Indeed, the rate of mortality is greater with Chloroform than with any other anæsthetic. But it is worthy of remark that every anæsthetic thus far discovered, has had its victims.

In view of the great mortality following the administration of chloroform, it becomes the profession to decide, with as much accuracy as possible, two very important questions:

1st. What conditions of the system—acquired or hereditary—and what peculiarities of constitution, forbid the administration of the agent?

2d. By what mode, if any, can this or any other anæsthetic, be safely administered?

As far as fatal cases now recorded, go to show, there can be no doubt that in attempting to settle definitely, the above questions, the profession are at sea, without compass or rudder.

It would appear that the *heart*, of all the organs, is the one most frequently assailed by the deadly influence of the anæsthetic poison. The *lungs* are the next, in frequency, to yield to its toxicological action. In many cases, the stomach, the brain and the tongue, by falling back, are the organs or parts, first to receive the shock of the toxic properties of the anæsthetic.

Deaths from chloroform may be either "sudden, gradual or secondary." But what are the causes of death from chloroform? Dr. Richardson asserts that when "a weakened and dilated right side of the heart, with enlarged hemorrhoidal veins, varicose veins of the lower extremities, and large, full, yet tense veins in the lower part of the body," is met with, we then have a patient to whom the administration is hazardous in the extreme. Another condition is where the kidneys are diseased; when the urine is loaded with albumen, and a uremic disposition to sleep manifested.

Fatty degeneration of the muscular structure of the heart forbids its employment. But the practical question turns upon our ability in determining accurately, the above pathological condition. Can it be done? Every sign and symptom upon which we may rely to determine the fact, may be wanting, and yet the patient may have fatty degeneration of the organ.

Hard drinkers, as a rule, should not be subjected to the anæsthesia of chloroform. The numerous cases of fatality resulting in such persons, should warn the Surgeon to withhold it.

Many, *particularly* obstetrical, practitioners, have employed small quantities of chloroform to induce *incomplete* anæsthesia, hoping to alleviate pain without the production of complete insensibility. Experience proves that partial anæsthesia is as dangerous to life, as when complete. We regret to know that many obstetricians habitually carry a bottle of chloroform, ready to employ it in every case of labor, under the impression that, when administered short of insensibility, it is safe and harmless. In many respects, anæsthetic agents are boons to suffering humanity. To the Surgeon, chloroform, even with its frequent fatal results, is indispensable. To the Obstetrician, in appropriate cases, it is invaluable. But it should be confined, in our opinion, strictly to cases imperatively demanding its employment; otherwise the risk and hazard of using it must frequently result in death; and when death does occur under such circumstances—when without it there was no danger—it is reprehensible and criminal in the first degree in one so reckless in its administration.

Having given these views, which we find announced by the highest authorities in the profession, we hope our readers will pardon us for suggesting the utmost caution, even in the employment of chloroform in doses sufficient for the induction of only partial anæsthesia. Many, we fear, in order to gain practice, resort to its use unnecessarily. Such motives are foreign to the principles and the true objects of our science.

